# Integrated Discourse Therapy After Glioblastoma: A Case Report of Face-To-Face and Tele-NeuroRehabilitation Treatment Delivery

**DOI:** 10.3389/fneur.2020.583452

**Published:** 2020-11-19

**Authors:** Lisa Milman, Emma Anderson, Katelyn Thatcher, Deborah Amundson, Chance Johnson, Morgan Jones, Louie Valles, Dale Willis

**Affiliations:** ^1^Language and Aphasia NeuroRehabilitation Lab, Department of Communicative Disorders and Deaf Education, Utah State University, Logan, UT, United States; ^2^Utah Education and Telehealth Network, Logan, UT, United States

**Keywords:** tumor, telerehabilitation, aphasia, language, therapy, discourse, case report

## Abstract

**Background:** Language and communication impairments are among the most frequently reported long-term behavioral consequences of brain tumor. Such deficits may persist long after a patient has been discharged from the hospital and can significantly impact return to work, resumption of prior social roles, and interpersonal relations, as well as full engagement in leisure activities. While considerable research has centered on identifying and describing communication impairments in brain tumor survivors, relatively little research has investigated language therapy for this population.

**Aims:** This report (1) reviews the literature and describes the language and cognitive-communicative profile of a 35-year-old man 6 years post glioblastoma excision with subsequent chemo- and radiation therapies; (2) presents cognitive-communication outcome data for this individual following an integrated discourse therapy; and (3) assesses treatment feasibility in face-to-face (F2F) and tele-neurorehabilitation (TNR) contexts.

**Methods:** A battery of tests and weekly conversation probes were administered to evaluate baseline performance and potential changes associated with F2F and TNR treatment delivery. Integrated Conversation Therapy (ICT) was administered across four alternating (F2F and TNR) treatment blocks over 2 months. ICT is a solution-focused discourse intervention that simultaneously targets word finding, sentence processing, and authentic patient-selected conversational interactions.

**Results:** Although the participant presented with long term-language impairments that were clinically distinct from stroke-associated aphasia, statistically significant post-treatment gains (>2 SEM) were evident following F2F and TNR treatment delivery on standardized measures of apraxia, discourse production, verbal memory, and self-ratings of discourse production, communication, and living with aphasia. While objective measures of treatment effect size (probes of CIU discourse data) were consistent across F2F and TNR delivery models, results of a satisfaction survey indicated a slight but statistically significant participant preference for TNR treatment delivery.

**Conclusions:** This study provides preliminary support for F2F and TNR delivery of ICT discourse intervention for glioblastoma survivors. It also highlights the need for more research specifically dedicated to language therapy for this population.

## Introduction

Studies report that at least 80% of BT survivors have cognitive and/or communication impairments at the time of initial diagnosis (1–6). These deficits can impact employment, social function, and overall quality of life long after patients have been discharged from the hospital (7–9). Literature reviews indicate that cognitive rehabilitation for BT survivors is feasible with the majority of individuals showing gains on measures of neuropsychological impairment (10–13), subjective ratings of cognitive function (11, 14), measures of independence (15, 16), and/or quality of life (17). Although this literature identifies language impairment as among the most common sequelae of brain tumor (6–8, 18), only a few studies have explicitly focused on language intervention. A recent review (19) identified several studies that provide preliminary evidence supporting language therapy for BT survivors. Some of these studies investigated broad rehabilitation outcomes and identified language/aphasia therapy as being a component of the program but did not provide specific details about the behavioral intervention (20–22). Other studies (23) focused on the language intervention but omitted key information associated with the medical history (e.g., tumor type/location) and/or biomedical treatments (e.g., chemotherapy, radiation). Only one study (23), targeting email use in individuals with severe cognitive impairment, included tele-neurorehabilitation (TNR) components (email with telephone follow-up).

This study adds a new case report of language TNR to this literature. BG, a glioblastoma survivor with a complex medical history and persistent non-fluent aphasia is unusual with respect to the extent of his initial tumor, the application of innovative chemotherapy, and his record of survivorship. Successful application of TNR technology would support BG, and similar patients, in continuing language treatment beyond the limited time spent in a medical/urban setting.

Based on prior research with the stroke population (24–26), we hypothesized that this treatment would be effective for several reasons. First, Integrated Conversation Treatment (ICT), targets language domains (word finding, sentence generation, and conversational interactions) that have been identified as being particularly vulnerable to brain tumor pathology (4, 8, 27). Second, in line with a large body of learning (28) and neurorehabilitation (25, 29–31) research, ICT explicitly trains *generalization* of isolated “part-task” (word/sentence production) to “whole-task” (functional communication) performance. Furthermore, ICT is a problem-based treatment approach that centers on authentic patient-selected communication goals. As such, successful acquisition of targeted treatment goals has the potential to have an immediate and positive impact on quality of life. While this is an important outcome for all patient populations, it is especially important for BT survivors who may be facing significant mental health/motivational issues associated with depression, anxiety, pain management, and/or malignant conditions with high mortality rates. Specific study objectives were to: (1) Present a detailed clinical history and cognitive-communication profile of a long term glioblastoma survivor; (2) Evaluate the efficacy of ICT; and (3) Assess feasibility of TNR by monitoring treatment progress and patient satisfaction during alternating face-to-face (F2F) and TNR treatment blocks.

## Case Description

### Medical History

BG, a 35-year-old right-handed monolingual English-speaking Caucasian male initially presented to the ER 6 years prior to this study after experiencing a “fuzzy feeling, mumbling, and aphasia” followed by seizure activity. A malignant (grade IV) 3 × 2.5 × 2 cm irregularly-shaped glioblastoma in the left frontal lobe extending deep to the insula was subsequently identified and excised followed by radiation and chemotherapy (temozolomide) without any subsequent long-term behavioral deficits. Approximately 10 months later, there was a recurrence of the tumor. Subsequent treatment included a second resection with placement of Gliadel wafers (chemotherapy), followed by additional radiation and chemotherapy (azixa trial lasting 18 months). The most recent MRI reports showed no evidence of tumor progression.

### Therapy History

Immediately after the second surgery, inpatient reports described BG as having mild right hemiparesis and moderate-severe expressive aphasia. Initial speech therapy targeted naming, left/right discrimination, reading short sentences, and legibly writing his full name, address, and date. Consistent with prior reports of glioma patients (32), BG demonstrated significant gains following surgery and was discharged from physical and occupational therapies after 2 months. However, speech therapy targeting speech motor control, language, processing speed, and verbal memory have been ongoing. Clinical reports indicate that BG has continued to make steady gains throughout this time.

### Social and Vocational History

At the time of the initial diagnosis, BG was working full-time as an electrician, pursuing a teaching degree, and living with his wife and two daughters (aged three and five) in a remote rural area. Since that time, BG has continued to live with his family (daughters now aged eight and ten), but has not returned to complete his teaching degree. Except for several months working as a laborer on a farm, he has remained unemployed.

## Timeline

A single subject (ABABA) multiple baseline design (Table 1) was applied to assess potential treatment effects with replication across behaviors. Treatment (8 weeks total) included four consecutive two-week blocks: (1) Treatment I in F2F context; (2) Treatment I in TNR context; (3) Treatment II in F2F context; and (4) Treatment II in TNR context.

**Table 1:** Timeline and study design showing assessment schedule in relation to treatment phase.

**Study phase**	**(A)**		**(B)**				**(A)**	**(B)**				**(A)**	
	**Pre-treatment**		**Treatment I conversations about Sports**				**Break**	**Treatment II conversations about daughters' Interests**				**Post-treatment**	
Timeline	1	2	1	2	3	4	1	1	2	3	4	1	2
(week)													
Treatment delivery			F2F	F2F	TR	TR		F2F	F2F	TR	TR		
Assessment	Screen												
	Test Battery											Test Battery	
	Probe	Probe	Probe	Probe	Probe	Probe	Probe	Probe	Probe	Probe	Probe	Probe	Probe
				Survey		Survey			Survey		Survey		

*F2F, face-to-face delivery; TNR, telerehabilitation delivery; Survey, Satisfaction Survey*.

## Therapy Program

### Assessment

#### Initial Screening and Selection of Language Treatment Goals

BG passed a hearing screening and reported no pre-tumor history of language, learning, psychiatric, or neurological impairment. During a structured interview using the Assessment for Living with Aphasia (33), BG communicated that he viewed talking and writing as his most significant communication impairments. He expressed frustration that he was no longer able to work and provide financially for his family. He also indicated that he missed being able to participate in complex conversations with his family and friends. BG said that he felt comfortable talking at home and in the community but was concerned that people did not respect him or view him as competent/intelligent. Following this interview, an assessment and treatment plan were developed based on BG's two self-selected treatment goals: to improve his ability to talk with his daughters about their interests (D) and to talk with friends about sports (S).

#### Pre- and Post-treatment Testing

Standardized measures (Supplementary Materials 1) were selected to evaluate a range of speech, language, and cognitive domains. We hypothesized that potential generalization effects would be evident on measures of communication (motor speech, naming, sentence generation, discourse production, and functional communication) but not on measures of non-verbal/visuo-spatial abilities. Statistically significant change (e.g., cut-off scores, z-scores, & SEM) was determined using published data/procedures (33–45). For the ALA (33) ratings and AphasiaBank discourse analyses (44, 45) experimental data were used to compute descriptive statistics (pre- and post-treatment means, SD, and SEM). A difference of ≥2 SEM was interpreted as statistically significant change.

#### Weekly Probe Testing

The primary outcome measure was production of fluent meaningful speech in the context of functional communication. Standardized coding procedures (45) were used to code/tally Correct Information Units/minute (CIUs/utterance). Weekly probes consisted of four 5-min topic-focused conversations. Each conversation was with a different conversational partner (CP). Two conversations (with CP1 & CP2) focused on sports and two conversations (with CP3 & CP4) focused on his daughters' interests. The participant and CP were told to talk about their respective topics for 5 min. No other information or structure was provided. Mean point-to-point coding reliability across conversations was 91% (range: 82–100%).

#### Satisfaction Survey

BG completed a 24-item Satisfaction Survey after each treatment block to evaluate treatment delivery in F2F and TNR contexts. The survey, developed specifically for this study, was based on content from other published TNR satisfaction surveys (46–49) and more general aphasia/communication rating scales (33, 43, 50, 51). Items focused on autonomy, communication, treatment, treatment location, and treatment technology/equipment.

### Integrated Conversation Therapy

#### Treatment Protocol

Sessions included three 15-minute activities: Word retrieval training, Topic-comment training, and Conversation practice (Supplementary Materials 2). To address impairments associated with motor planning and processing speed, at the start of each activity BG was reminded to speak as clearly as possible. If an utterance was dysfluent and/or unintelligible during any of the tasks he was prompted “to stop, breathe, and think about what he wanted to say.”

Sessions began with word retrieval training modeled after semantic feature analysis (52, 53). Words (1–5 syllable nouns) were chosen by the participant in consultation with the clinician. Stimuli included 40 words related to sports and 38 words related to his daughters' interests (Supplementary Materials 3). The two sets of words were further divided into two balanced (frequency, length, phonetic complexity) subsets used for F2F and TNR delivery.

Topic-comment training, modeled after Response Elaboration Training (RET) (54, 55) trained word retrieval and fluency (CIUs/utterance) of target words in the context of sentences. The clinician produced ten novel topic-related comments/questions (e.g., I watched the super bowl last night) to elicit a spontaneous comment/question from BG (e.g., What team were you rooting for?).

Conversation practice, also modeled after RET, targeted retrieval of trained words and sentences in the context of topic-focused conversations. BG selected a topic related to either sports (Sports Conversation treatment) or his daughters' interests (Daughters' Interests Conversation Treatment).

#### Dosage and Setting

Four 45-min sessions per week for 8 weeks were conducted in a quiet setting. F2F sessions occurred in a clinical suite with BG sitting opposite the clinician. For TNR sessions, BG participated from his home and communicated with the clinician (located at the clinic) by computer.

#### Tele-Neurorehabilitation System Architecture

TNR sessions were run via internet (5 mb/s incoming signal; 1 mb/s outgoing signal) in real time using commercially available CISCO jabber videoconferencing system software. Clinicians used a CISCO SX10 videoconferencing system integrated with a remote-controlled HD (768 × 448) video camera, a CISCO CTS-QS C20 microphone, and a widescreen (30″) Samsung display. The participant accessed the system from home via Digis broadband internet using a system-configured widescreen (15.6″) Dell Inspiron 1545 (2GHz dual-core, 1MB, 800 MHz processor; 3GB RAM) laptop computer equipped with a Microsoft LifeCam Cinema 720p HD (30 frames/s) webcam and integrated microphone. Sessions were recorded and stored on an established, controlled-access, secure state education/healthcare server.

#### Treatment Fidelity

Two graduate students supervised by an ASHA certified SLP (study authors) administered the therapy. An anlysis of treatment fidelity (sequential completion of treatment steps as specified in Supplementary Materials 2) showed 93% concordance between actual and specified study procedures.

### Outcomes

#### RQ1 Baseline Cognitive-Communicative Profile

Performance (Supplementary Materials 1) was above or within the normal range on many measures of apraxia (ABA-2 subtests), comprehension/production of single words and sentences (WAB-R, NAVS), verbal memory (Digit Span), verbal learning (CVLT-2), and non-verbal problem solving (RCPM). Mild impairments were evident on diadochokinetic rate (ABA-2), higher-level naming (BNT), general measures of aphasia (WAB-R), and cognitive-communicative ability (SCCAN). BG's most significant (moderate-severe) impairments were identified on production of words of increasing length (ABA-2), intrusion and false positive errors on delayed recall of word lists (CVLT-2), and discourse production (WAB-R fluency rating & AphasiaBank).

In contrast to relatively preserved production of isolated words and sentences, discourse production was characterized by an atypical fluency pattern, alternating between phases of normal fluent speech, slow effortful groping articulation, and occasional low volume rushes of unintelligible speech. Marked word finding behaviors (false starts, fillers, pauses, phonemic, and semantic paraphasias, circumlocution), paragrammatism (simplified perseverative structural patterns with functor errors), as well as reduced information content were also more pronounced during connected speech than in production of isolated words and sentences.

Using the WAB-R criteria, BG was classified as having a mild (AQ = 89.2) anomic aphasia. BG self-described his language impairment as significantly impacting his participation in daily activities and his overall quality of life.

#### RQ2 Treatment Efficacy

##### Acquisition of target behavior

**Figure 1:**
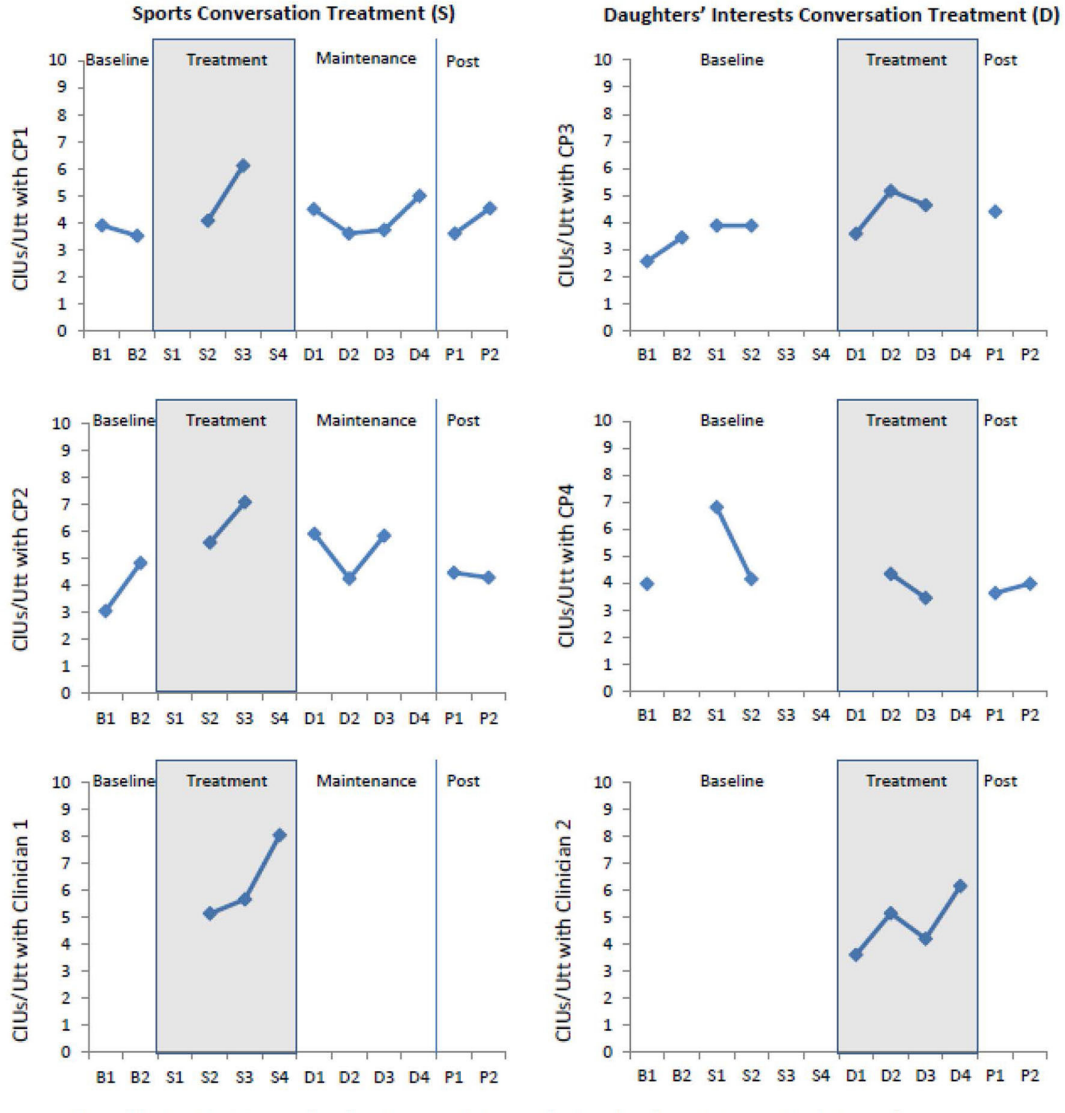
CIU's/utterance produced by BG during probes (top four graphs) and during treatment sessions (bottom two graphs) with six conversation partners (CP1, CP2, Clinician 1, CP3, CP4, and Clinician 2) during Sports and Daughters' Interest conversations throughout baseline, treatment, and post-treatment phases. Missing data from probes (top four graphs) were due to missed appointments by designated conversational partners. To supplement this missing information information, data from conversations with clinicians during treatment session (bottom two graphs) are also included.

Production of CIUs/utterance (Figure 1) increased during treatment with five of the six conversational partners. Computation of a weighted d statistic (56) using the probe data (top four graphs) revealed a small overall treatment effect size (*d* = 0.9) (Table 2).

**Table 2:** Treatment effect size with each conversation partner (CP).

	**BG sports conversations**	**BG sports conversations**	**BG daughters interests' conversations**	**BG daughters interests' conversations**	**TOTAL d (sum of weighted d/ sum of observations)**
	**(with CP1)**	**(with CP2)**	**(with CP3)**	**(with CP4)**	
d	1.63	0.8	1.56	−0.7	0.91
Observations	8	7	5	5	25
Weighted d	13.05	5.62	7.79	−3.7	22.8

##### Pre- and post-treatment standardized test battery (Supplementary Materials 1)

Significant change (≥ 2 SEM) was evident on measures of apraxia (ABA-2 Diadochokinetic Rate & Increasing Word Length), verbal memory (Digit Span Forward & CVLT-2 subtests: Free Recall List B, Short-Delay Free Recall, and Long-Delay False Positives), self-ratings of communication (CETI), self-ratings of living with aphasia (ALA Participation Domain, ALA Personal Domain), and discourse production (AphasiaBank Free Speech). In addition, BG showed a change in diagnostic classification on the BNT (pre-treatment = mild impairment; post-treatment = normal) and the Total Intrusions score on the Delayed Recall Test of the CVLT (pre-treatment = impaired; post-treatment = normal).

#### RQ3 Feasibility of F2F vs. TNR Treatment Delivery

##### BG attended all sessions

Treatment data (Figure 1) suggest a relatively stable learning curve, regardless of whether the intervention was administered in F2F or TNR contexts. Ratings from the Satisfaction Survey ranged from 3 to 5 (maximum possible = 5). Of the 21 items, ten had identical ratings for F2F and TNR conditions, nine had higher ratings for the TNR condition, and two had higher ratings for the F2F condition. Collapsing across the 21 items and both treatment replications (Sports and Daughters' Interests), results of a paired sample t-test revealed a small but statistically significant difference between F2F (mean rating = 4.57, SEM = 0.08) and TNR (mean rating = 4.76, SEM = 0.07) satisfaction ratings (t = −2.24, df = 41, *p* < 0.03).

## Discussion

### Cognitive-Communicative Profile

BG performed in the mildly impaired to normal range on many basic measures of cognitive-communicative function. Yet more in depth evaluation revealed moderate-severe impairments affecting motor speech, language, verbal working memory, functional communication, and quality of life. On the WAB-R, BG was classified as having a mild anomic aphasia. However, his communication differed from a classic anomic pattern. The most notable deviations included: fluctuating production (affecting fluency and information content); a marked discrepancy between production of isolated words and sentences vs. spontaneous connected speech; and involvement of more diffuse behavioral signs (specifically motor speech production and verbal working memory). While not characteristic of classic anomia, BG's performance was similar to reports of other BT survivors (27, 57, 58) and to cases of dynamic aphasia associated with BT and/or atypical aphasia resulting from subcortical pathology (59–61). As hypothesized elsewhere (8, 27, 57, 58, 62, 63), these distinct behavioral effects are likely tied to the involvement of widely distributed subcortical networks (associated with speech production, language, working memory, and executive functions) and the unique pathophysiology precipitated by the tumor and/or medical treatments (neurosurgery, chemotherapy, and radiation therapy).

### Response to Integrated Conversation Treatment

BG made statistically significant gains on the primary outcome measure (CIUs/Utterance in topic-focused conversations) and on standardized measures of apraxia, discourse production, verbal working memory, functional communication, and quality of life. Responses to the Satisfaction Survey and other anecdotal comments also indicate that BG and his wife perceived the intervention as helpful. For instance, BG described the intervention as being particularly effective relative to prior language therapy and commented that the intervention played an important role in his decision and ability to return to work shortly after this study was completed.

Given that the intervention targeted isolated language production tasks (motor speech control, word finding, sentence generation) *and* generalization of training to authentic everyday conversational interactions, the gains observed across a range of language and communication measures were not surprising. While changes in verbal memory were not anticipated, these findings are consistent with prior research linking language production with verbal working memory (64, 65).

BG's most significant changes were on measures of spontaneous speech, verbal memory, and functional communication; whereas in previous ICT studies (of post-stroke aphasia) greatest gains were seen on standardized measures of naming (BNT) and on a general aphasia battery (WAB-R). Several factors likely contributed to these differences. First, the etiology of BG's aphasia likely contributed to not only differences in baseline performance but also to differences in his responsiveness to treatment. A closely related issue centers on assessment. BG performed at or near ceiling on standardized language measures (WAB-R & BNT) used in previous studies. As recently indicated in the BT survivor literature (57, 66–68), it is likely that these measures (developed for other clinical groups), were not sufficiently sensitive to capture changes in BG's ability level.

It is also likely that modifications of our treatment approach contributed to differences in the observed outcomes and increased carry over to everyday communication. First, to increase personal relevance of ICT, we asked BG to choose not only treatment stimuli (as in previous studies with stroke patients) but also the discourse topic. Personal relevance has been identified as a critical variable in facilitating neuroplasticity (30), positive outcomes in aphasia treatment research (69), and increased language production in cases of dynamic aphasia (60).

A second important modification of our treatment approach was the shift in whole-task training from a picture-based descriptive discourse task (as done in earlier studies) to practiced engagement in authentic conversational interactions. Consistent with previous findings in the aphasia treatment literature (31, 70), we observed substantial variability in performance (and generalization) across discourse genres, topics, and even conversational partners. Notably, the greatest change in performance was observed on the trained conversation task. Results of this study, therefore, add to growing evidence highlighting the multifaceted specificity of discourse processing, and the benefit of directly targeting conversation in therapy (26, 31).

### Comparison of Face-To-Face (F2F) and Tele-Neurorehabilitation (TNR) Intervention

BG, showed a relatively stable learning curve (Figure 1) acrossF2F and TNR delivery. With respect to the Satisfaction Survey, results indicated a small but statistically significant preference for the TNR condition. In fact, the one negative comment written on the survey was BG's dissatisfaction with the time that it took him to drive to therapy. Collectively these data add to an existing literature supporting the feasibility of TNR and the potential for comparable patient satisfaction with both treatment delivery models (11).

### Limitations and Future Research Priorities

These findings reflect data from a single case, therefore more research is needed before inferences can be made to a larger group. Also, we began with F2F treatment (standard delivery model) to ensure that ICT was an effective therapy for BG, before assessing the potential feasibility of TNR delivery. Use of an alternating design (counterbalancing the order of F2F and TNR conditions) and extending the duration of the treatment phase would provide more definitive information about the relative efficacy of F2F vs. TNR delivery. Also, exploring variable dosage levels (e.g., number of sessions/week; adding “booster” maintenance sessions) would support more flexible clinical applications.

## Conclusions

BG's cognitive-communication profile and response to intervention differed from other individuals who have participated in the ICT protocol. Nonetheless, statistically and clinically significant change were observed on measures of apraxia, spontaneous speech production, verbal working memory, and quality of life. These preliminary findings add to a growing literature suggesting that both conventional and alternate delivery models, such as TNR, may be effective and practical options for brain tumor survivors.

## Data Availability Statement

The original contributions presented in the study are included in the article/Supplementary Materials, further inquiries can be directed to the corresponding author/s.

## Ethics Statement

The studies involving human participants were reviewed and approved by Utah State University Institutional Review Board. The patients/participants provided their written informed consent to participate in this study.

## Author Contributions

LM and EA conceptualized the study. EA, KT, DA, and LM administered the intervention. LM, EA, CJ, and MJ contributed to the data analyses. LV and DW were responsible for the TNR technology and videoconferencing. All authors contributed to the article and approved the submitted version.

## Conflict of Interest

The authors declare that the research was conducted in the absence of any commercial or financial relationships that could be construed as a potential conflict of interest.

## Supplementary Material

Supplementary Material 1 Pre- and Post-testing results on Standardized Tests.

Supplementary Material 2 Appendix A Treatment Protocol.

Supplementary Material 3 Appendix B Treatment Stimuli.

Supplementary Material 4 CARE Checklist.
