# Supplementary material for: Integrated Discourse Therapy After Glioblastoma: A Case Report of Face-To-Face and Tele-NeuroRehabilitation Treatment Delivery
Source: Front Neurol. 2020 Nov 19;11:583452. doi: 10.3389/fneur.2020.583452 (PMC7710897; doi:10.3389/fneur.2020.583452)
Supplement: Supplementary Material 1 — Pre- and Post-testing results on Standardized Tests. [file Data_Sheet_1.docx]

| **Supplementary Materials 1**  *Pre- and post-treatment performance on standardized measures with means (M) and standard error of measurement (SEM)* | | | |
| --- | --- | --- | --- |
| **Measure** | **SEM** | **Pre-Treatment** | **Post-Treatment** |
| **Criterion and Norm Referenced Tests**  Motor Speech Screen (max = 66) | NA | 62 (mild impairment) | 60 (mild impairment) |
|  |  |  |  |
| Apraxia Battery for Adults-2^a^  (cut-off scores) |  |  |  |
| 1 Diadochokinetic Rate (normal ≥ 26) | 2.27 | 15 (mild impairment) | 35 (normal) ^*^ |
| 2A Increasing Word Length (normal ≤ 1)  2B Increasing Word Length (normal ≤ 1) | 0.04  0.07 | 8 (severe impairment)  (NA) | 1 (normal) ^*^  2 (mild impairment) ^*^ |
| 3A Limb Apraxia (normal ≥ 44) | 0.65 | 50 (normal) | 50 (normal) |
| 3B Oral Apraxia (normal ≥ 44) | 0.40 | 50 (normal) | 50 (normal) |
| 4 Utterance Time (normal ≤ 15) | 1.25 | 11 (normal) | 17 (mild impairment)^*^ |
| 5 Repeated Trials (normal ≥ 28) | 0.08 | 30 (normal) | 30 (normal) |
| 6 Inventory of Articulation (max=15) | 0.36 | 7 characteristics of apraxia | 7 characteristics of apraxia |
|  |  |  |  |
| Western Aphasia Battery-Revised^b^ |  |  |  |
| Aphasia Classification |  | Anomic | Anomic |
| AQ (M = 99.6) | 4.59 | 89.2 (mild impairment) | 89.2 (mild impairment) |
| Information Content (M = 10) | 0.67 | 9.0 (mild impairment) | 9.0 (mild impairment) |
| Fluency (M = 10)) | 0.71 | 6.0 (moderate impairment) | 6.0 (moderate impairment) |
| Auditory Comprehension (M = 10) | 1.94 | 10.0 (normal) | 10.0 (normal) |
| Repetition (M = 9.9) | 0.59 | 9.8 (normal) | 9.6 (normal) |
| Naming (M = 9.8) | 0.60 | 9.8 (normal) | 10.0 (normal) |
|  |  |  |  |
| Boston Naming Test (M = 55.8) | 1.66 | 51 (mild impairment) | 53 (normal) |
|  |  |  |  |
| NAVS (percent correct) |  |  |  |
| Verb Naming (M = 99.6) | 0.3 | 100 (normal) | 100 (normal) |
| Verb Comprehension (M = 100) | 0.1 | 100(normal) | 100 (normal) |
| Argument Production (M = 99.4) | 0.6 | 98 (borderline normal) | 98 (borderline normal) |
| Sentence Production (M = 99.6) | 0.6 | 100 (normal) | 100 (normal) |
| Sentence Comprehension (M = 99.6) | 0.6 | 100(normal) | 100 (normal) |
|  |  |  |  |
| Digit Span  Forward (M = 6)  Backwards (M = 5) | 1  1 | 6 (normal)  4 (normal) | 9 (normal)*  5 (normal) |
|  |  |  |  |
| CVLT-2 |  |  |  |
| T1-5 Free Recall Total (M = 47.9) | 5.07 | 45(normal) | 53 (normal) |
| Free Recall List B (M = 5.1) | 1.36 | 4 (normal) | 7 (normal)* |
| Short-Delay Free Recall (M = 10.0) | 1.57 | 6 (borderline) | 14 (normal)* |
| Short-Delay Cued Recall (M = 12) | NA | 8(borderline) | 13(normal) |
| Long-Delay Free Recall (M= 10.2) | 1.35 | 10 (normal) | 11(normal) |
| Long-Delay Cued Recall (M = 12.5) | NA | 10 (normal) | 13(normal) |
| Total Intrusions (M = 5.0) | 3.54 | 12 (impaired) | 9 (normal) |
| Total Repetitions (M = 4.6) | 4.34 | 2 (normal) | 6 (normal) |
| Long-Delay Hits (M = 14.4) | 0.85 | 16(normal) | 16 (normal) |
| Long-Delay False Positives (M = 2.8) | 2.03 | 10 (impaired) | 1 (normal)* |
|  |  |  |  |
| RCPM (M = 34.9) | 0.56 | 34 (normal) | 36 (normal) |
|  |  |  |  |
| SCCAN (M = 92) | 3.75 | 83 (mild impairment) | 86 (mild impairment) |
|  |  |  |  |
| **Patient ratings of**  **Communication & QOL** | | |  |
| CETI^c^ (max = 68) | 4.12 | 62.5 | 91.25* |
|  |  |  |  |
| ALA ratings^c^ (max = 4) |  |  |  |
| Total | 0.28 | 3.18 | 3.63 |
| Aphasia Domain | 0.61 | 2.90 | 3.20 |
| Participation Domain | 0.31 | 3.12 | 3.74* |
| Environment Domain | 0.40 | 3.38 | 3.38 |
| Personal Domain | 0.18 | 3.36 | 3.77* |
| Wall Question (single item, SEM NA) | NA | 3.00 | 3.50 |
|  |  |  |  |
| **AphasiaBank Discourse Battery^c^**  **(mean CIUs/utterance)** |  |  |  |
| Total | 1.08 | 6.5 | 8.1 |
| Free Speech | 1.08 | 3.6 | 7.1* |
| Picture Description | 0.71 | 6.7 | 7.9 |
| Cinderella narrative | 0.97 | 8.3 | 8.8 |
| Procedural discourse | 0.69 | 7.3 | DNT |

* Indicates significant increase (> 2 SEM) from baseline

*Notes.* Scoring and interpretation based on information provided in test manuals: Motor Speech Screen (Duffy, 2013); Apraxia Battery for Adults-2 (ABA-2, Dabul, 2000); Western Aphasia Battery-R (WAB-R, Kertesz, 2007); Boston Naming Test (BNT, Kaplan et al., 2001; reliability coefficient from Spreen & Strauss, 1998); Northwestern Assessment of Verbs and Sentences (NAVS, Thompson, 2011; reliability coefficient from Cho-Reyes & Thompson, 2012); AphasiaBank Discourse Battery (MacWhinney et al., 2011; reliability coefficient for CIU analyses from Nicholas & Brookshire, 1993); Scales of Cognitive and Communicative Ability for NeuroRehabilitation (SCCAN, Milman & Holland, 2012); Raven’s Colored Progressive Matrices (RCPM, Raven, Raven, & Court, 1998; reliability coefficient from Spreen & Strauss, 1998); Digit Span (Lezak, 1995); California Verbal Learning Test-2 (CVLT-2, Delis, Kramer, Kaplan, & Ober, 2000); Communicative Effectiveness Index (CETI, Lomas et al., 1989); Assessment for Living with Aphasia (ALA, Kagan et al., 2010; reliability coefficients from Simmons-Mackie et al., 2014a).
