# Supplementary material for: Integrated Discourse Therapy After Glioblastoma: A Case Report of Face-To-Face and Tele-NeuroRehabilitation Treatment Delivery
Source: Front Neurol. 2020 Nov 19;11:583452. doi: 10.3389/fneur.2020.583452 (PMC7710897; doi:10.3389/fneur.2020.583452)
Supplement: Supplementary Material 2 — Appendix A Treatment Protocol. [file Data_Sheet_2.docx]

**Supplementary Materials 2: Appendix A. Treatment Protocol**

Treatment included three consecutive therapy tasks: Word Retrieval Training, Topic-Comment Training, & Conversation Practice. At the start of each task BG was reminded to “Stop, Breathe, and think before speaking.”

*Word Retrieval Training*

For each item, a semantic feature analysis template (Boyle, 2004) was presented and BG was asked to name the target picture. Next, regardless of success on the initial naming attempt, BG was asked to say and then write down a) the semantic category (“What kind of thing is it?”), b) an association (“Tell me something about it”), c) the first sound (What is the first sound?”), and then d) the name of the item. If BG was unable to produce any of the targets after prompts a-d (category, association, sound, or name) a multiple choice option was provided (*Is it a piece of equipment or clothing?*). If an utterance was dysfluent at any point in this procedure, BG was prompted to: “*Stop, breathe, and think about what you want to say. Then say it again, nice and slowly.”*

*Topic-Comment Training*

The topic-comment intervention was modeled after Kearn’s (1985) Response Elaboration Training. The clinician initiated each spoken exchange with a topic-focused comment/question (e.g. “*Did you watch the opening ceremonies of the Olympics?”*). This was followed by BG’s independently generated topic-focused spoken response (“*yes, I saw it”*). The clinician then repeated and prompted an expansion of this response *(“Oh, so you watched the opening ceremonies last night, what was your favorite part?)*. After BG answered the question (“*I liked seeing the athletes”*), the clinician repeated and expanded the client’s response by combining it with his original utterance *(“So you watched the opening ceremonies last night and your favorite part was seeing all the athletes – is that right?”)*. Once the client agreed with the clinician’s recast of his utterance, he was asked to repeat the expanded utterance fluently. If an utterance was dysfluent at any point in this procedure, BG was prompted to: “*Stop, breathe, and think about what you want to say. Then say it again, nice and slowly.”*

*Conversation Practice*

BG was asked if there was something he wanted to talk about related to sports (during treatment phases I and II) or his daughters’ interests (during treatment phases III and IV). If he did not have a specific topic of choice the clinician suggested some options (e.g. “Do you want to talk about your fishing trip or the football game?”). Prior to initiating the conversation, the clinician said “*Remember to stop, breathe, & think before you start talking. If you say something that’s unclear, I’ll just tell you that I didn’t understand and ask you to repeat it.”* If BG produced an utterance that was dysfluent at any point, the clinician requested a clarification (*“I am sorry I didn’t understand that. Can you say it again?”)*. If the utterance was still dysfluent, the clinician initiated the same modified Response Elaboration Training (Kearns, 1985) cueing protocol described above for topic-comment training.
