# Supplementary material for: Integrated Discourse Therapy After Glioblastoma: A Case Report of Face-To-Face and Tele-NeuroRehabilitation Treatment Delivery
Source: Front Neurol. 2020 Nov 19;11:583452. doi: 10.3389/fneur.2020.583452 (PMC7710897; doi:10.3389/fneur.2020.583452)
Supplement: Supplementary Material 3 — Appendix B Treatment Stimuli. [file Data_Sheet_3.docx]

**Supplementary Materials 3:**

**Appendix B. Vocabulary used for Word Retrieval Training for each treatment block**

| Sports-F2F | Sports-TR | Daughter’s Interests-F2F | Daughter’s Interests-TR |
| --- | --- | --- | --- |
| jig | key | bunt | rings |
| punt | lures | sludge | chores |
| nymph | assist | Elsa | Anna |
| blitz | waders | satyr | salute |
| spoons | jump shot | front tuck | third base |
| angler | end zone | cartwheel | round off |
| Keeton | alley-oop | Grandpa | Granny |
| turnover | crossover | fair ball | balance beam |
| free throw | wide receiver | Miss Lou | Mrs. Barton |
| field goal | running back | Sponge Bob | Hungry Hippos |
| third down | Nick Vigil | Duty | Merida |
| shot clock | three point line | recess | Somersault |
| Kapernick | Jim Harbaugh | tumbling | tumbling gym |
| travelling | flagrant foul | leotard | salt lick block |
| game warden | yellow perch | infielder | back handspring |
| fishing Pole | free agent | Taylor Swift | Tim McGraw |
| spinning rod | cutthroat trout | Seth Sorenson | Librarian |
| hot & tots | Kevin Durrant | Kendra Sorenson | Fan Boy and Chum Chum |
| incomplete pass | technical foul |  | Breadwinners |
| New York Yankees | Weston Reservoir |  | macaroni and cheese |
